# The Sleeping Sickness Bureau

**Published:** 1909-03-13

**Authors:** 


### THE SLEEPING SICKNESS BUREAU.

Recently a sleeping-sickness bureau, under the charge of a director, was started in London, its chief aims and objects being to collect all the available literature together, to condense and sift this out, and then to publish it in an easily assimilable form for those interested in or working on the subject. The committee were fortunate to be able to secure the services of Dr. A. G. Bagshawe, of the Uganda medical staff, as director, and this gentleman has now brought out the first bulletin of the Bureau. The plan of the publication is a good one, a short summary or synopsis of the different papers—in this case the treatment of trypanosomiasis, the biological accommodation of trypanosomes to chemo-therapeutic agents, and the treatment of experimental animals—is given, and a number attached to the name of each author refers to an alphabetically arranged bibliography at the end of the book. By running one's eye over the different pages one quickly gets a very complete account of the whole subject, and then the author in the conclusion sums up the chief points. There are many interesting features in treatment to be found in the bulletin; thus the atoxyl-proof strains of the parasites, the antimony treatment, the combined atoxyl and mercury treatment are dealt with among other matters. Dr. Bagshawe concludes that the employment of atoxyl or any other trypanocide by itself has ceased to be justifiable; combined therapy is more useful, and if one or other produces no effect then alternating ones should be tried. In the past there has been a strong tendency to press the dose of some of these drugs too far, and untoward symptoms have from time to time appeared. The warning of the cases of blindness after the use of atoxyl, mentioned here and elsewhere, should make people more careful.

